# Comprehensive Analysis of Influenza Viruses’ Trends in Italy: Insights from a Nationwide and Regional Perspective

**DOI:** 10.3390/idr17020020

**Published:** 2025-02-27

**Authors:** Francesco Branda, Nicola Petrosillo, Dong Keon Yon, Massimo Ciccozzi, Fabio Scarpa

**Affiliations:** 1 Unit of Medical Statistics and Molecular Epidemiology, Università Campus Bio-Medico di Roma, 00128 Rome, Italy; m.ciccozzi@unicampus.it; 2 Infection Prevention & Control/Infectious Disease Service, Fondazione Policlinico Universitario Campus Bio-Medico, 00128 Rome, Italy; 3 Center for Digital Health, Medical Science Research Institute, Kyung Hee University College of Medicine, Seoul 02448, Republic of Korea; yonkkang@gmail.com; 4 Department of Biomedical Sciences, University of Sassari, 07100 Sassari, Italy; fscarpa@uniss.it

**Keywords:** human metapneumovirus (hMPV), influenza A(H3N2), influenza A(H1N1)pdm2009, influenza B, respiratory syncytial virus (RSV), rhinovirus, adenovirus, human bocavirus (HBoV), parainfluenza virus, disease surveillance, public health communication, respiratory viruses, epidemiological monitoring, real-time data sharing

## Abstract

**Background**. Influenza remains a significant public health issue, with seasonal trends varying across regions. This study provides a comprehensive analysis of influenza virus trends in Italy, leveraging epidemiological and virological data from the Istituto Superiore di Sanità (ISS). The primary objective is to assess influenza activity at both national and regional levels, highlighting variations in incidence rates and viral subtype circulation during the 2023/2024 season. **Methods**. We conducted a systematic approach to data collection, processing, and visualization, utilizing influenza surveillance data from ISS. Incidence rates, subtype distribution, and co-circulating respiratory viruses were analyzed to identify key trends. **Results**. Our findings reveal a significant increase in influenza cases during the 2023/2024 season, with incidence rates surpassing pre-pandemic levels. Notably, changes in the circulation of influenza A(H3N2) and influenza B were observed, alongside the presence of other respiratory viruses such as RSV and rhinovirus. **Conclusions**. This study underscores the importance of real-time surveillance, transparent data sharing, and advanced visualization tools in guiding public health responses. By integrating lessons from COVID-19, we highlight the necessity of standardized surveillance frameworks to enhance preparedness for future seasonal outbreaks and potential pandemics.

## 1. Introduction

The recent spread of human metapneumovirus (hMPV) in China [1], five years after the COVID-19 pandemic, alongside reports of the so-called “mystery disease” in the Democratic Republic of the Congo (DRC) [2], underscores the ongoing challenges in global health crisis management and science communication. These two events, though distinct in geography and context, highlight recurring themes: the importance of robust health response mechanisms, effective and transparent communication strategies, and the delicate balance between vigilance and avoiding unnecessary alarm. In an increasingly interconnected world, where information spreads as rapidly as pathogens, it is imperative that health crisis communication remains evidence-based, adaptable, and resistant to sensationalism, fostering trust among populations while facilitating swift and effective responses.

Respiratory viruses represent a significant burden on global public health. Influenza viruses, in particular, have been a persistent threat for centuries, causing seasonal epidemics and occasional pandemics. The 1918 influenza pandemic, for example, caused around 50 million deaths worldwide [3]. The 2009 H1N1 pandemic highlighted the rapid global spread of new influenza strains and the need for robust surveillance systems [4]. Influenza viruses are characterised by high mutation rates, allowing them to evade immune responses and necessitating annual updates of vaccine formulations [5]. In addition to influenza, several other respiratory viruses contribute significantly to the overall disease burden, particularly among young children, the elderly, and immunocompromised individuals. Respiratory syncytial virus (RSV) is a major cause of bronchiolitis and pneumonia in infants, resulting in substantial morbidity and hospitalization rates each year [6]. Adenoviruses are responsible for a wide spectrum of respiratory illnesses, ranging from mild cold-like symptoms to severe pneumonia, and are particularly problematic in closed communities such as military barracks and daycare centers [7]. Human bocavirus (HBoV), first identified in 2005, is increasingly recognized as an important pathogen in pediatric respiratory infections, often circulating with other viruses and complicating diagnosis and management [8]. In addition, other endemic coronaviruses (HCoV-229E, HCoV-NL63, HCoV-OC43, HCoV-HKU1) continue to circulate seasonally, causing upper respiratory tract infections that can be severe in high-risk populations [9].

Among these well-established pathogens, hMPV has attracted considerable attention in early 2025 [10]. It is a respiratory virus within the family *Paramyxoviridae* and the genus *Metapneumovirus*, was first identified in 2001 and has since emerged as a significant pathogen responsible for acute respiratory infections. Particularly vulnerable populations include children, the elderly, and individuals with compromised immune systems. The virus, a single-stranded negative-sense RNA pathogen, primarily infects the upper and lower respiratory tracts. Its clinical manifestations range from mild symptoms such as the common cold to severe conditions like bronchiolitis and pneumonia. Transmission occurs predominantly through respiratory droplets, though contaminated surfaces can also facilitate its spread. Symptoms of hMPV infection typically include fever, cough, difficulty breathing, and wheezing, with severe cases often requiring hospitalization, especially among high-risk groups such as infants, the elderly, and those with underlying comorbidities. Despite the absence of specific antiviral treatments, most cases respond well to symptomatic management. The recurrent emergence of respiratory pathogens like hMPV serves as a critical reminder of the need for sustained investment in public health infrastructure, proactive disease surveillance, and ongoing research into potential therapeutic and preventive measures [11,12].

The recurrent emergence of respiratory pathogens serves as a critical reminder of the need for sustained investment in public health infrastructure, proactive disease surveillance, and ongoing research into potential therapeutic and preventive measures. Surveillance systems play a key role in detecting outbreaks early, monitoring the spread of viruses, and informing public health interventions. For example, the Global Influenza Surveillance and Response System (GISRS), established by the World Health Organization (WHO), has been instrumental in tracking influenza viruses globally and guiding vaccine development [13]. However, the effectiveness of surveillance systems depends on timely and transparent data sharing [14]. The COVID-19 pandemic demonstrated both the power and challenges of real-time data exchange. Rapid sharing of epidemiological data helped guide decisions on containment measures, testing, and vaccine development, but it also highlighted the need for standardized frameworks for data collection and dissemination [15].

This paper offers a comprehensive snapshot of influenza in Italy through a systematic analysis of epidemiological and virological data extracted from bulletins published by the Istituto Superiore di Sanità (ISS). By presenting a graphical tool developed in collaboration with Il Sole 24 Ore, we provide an intuitive and immediate visualization of influenza trends, ensuring clear and effective communication. Although predictive models are valuable for identifying trends, accurately predicting the exact trajectory of a viral outbreak remains a complex challenge. For this reason, relying on robust data-driven analyses is essential to guide effective public health responses. The COVID-19 pandemic demonstrated the critical need for transparent data sharing to improve preparedness for future public health emergencies.

## 2. Materials and Methods

In Italy, our research group has undertaken a comprehensive effort to analyze and share data from the ISS on hMPV and other respiratory viruses, ensuring that these resources are easily accessible to the scientific community. The initiative involves collecting detailed information on the number of clinical samples analyzed, the genetic sequences obtained, and the detection of respiratory viruses. While data on respiratory viruses cover the 2003–2024 influenza seasons, specific data on hMPV have been systematically collected and made available since the 22022–2023 season. As part of this effort, data extracted from ISS bulletins, originally available in PDF format, were converted to machine-readable formats. This transformation improves usability, enabling more efficient analysis, integration with other datasets, and broader accessibility for research and public health applications. A key component of this initiative was the creation of a centralized, comprehensive database designed to systematically store and manage critical information on respiratory viruses circulating in Italy. This database not only improves the effectiveness of monitoring and research, but also serves as a crucial resource for real-time surveillance, enabling rapid detection of emerging trends and promoting ongoing collaboration among the scientific and health communities. By providing standardized, high-quality data, this project aims to provide researchers and policymakers with the tools they need to develop timely, evidence-based strategies to address current and future public health challenges.

**Figure 1:**
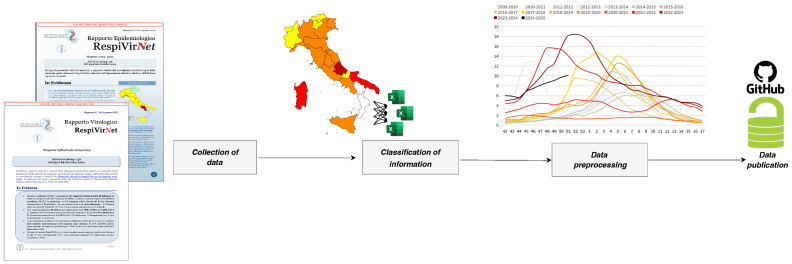
Schematic overview of the key steps to build the dataset.

Figure 1 summarized the data production process covering from the digitalization to the release of the dataset. The first step, i.e., Collection of data, was carried out through manual extraction of information from the weekly PDF bulletins published by RespiVirNet [16], the Italian surveillance system of influenza-like illnesses and respiratory viruses, coordinated by ISS with the support of the Ministry of Health, which monitors influenza-like illnesses and respiratory viruses throughout the country.

**Figure 2:**
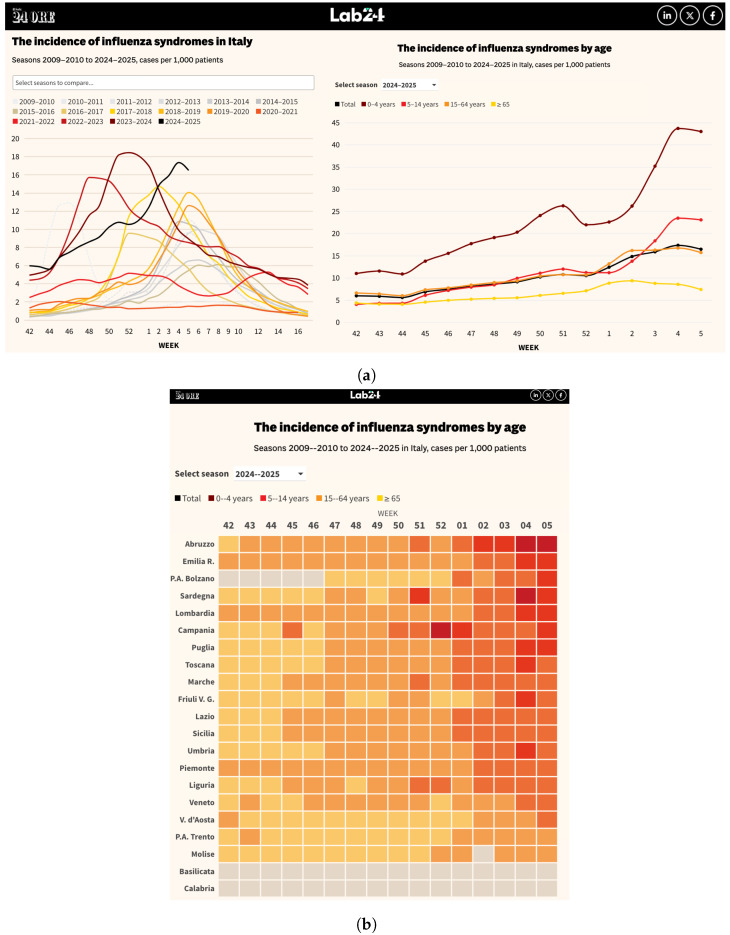
Example of dataset usage [17]. (**a**) Weekly trend of ILI incidence at the national level and across different age groups. (**b**) Weekly ILI incidence per region.

The bulletins contained data on the types of viruses identified (influenza A and B, RSV, adenovirus, etc.), incidence of illness in different age groups, geographic distribution on a regional basis, and time periods defined by epidemiological weeks. This information was collected manually because bulletins were available in unstructured formats, requiring special care to avoid errors during conversion. Once the collection was completed, the information was organized through a classification process that divided the data into main categories, including epidemiological data (incidence rate of ILI by region and age group), virological data (test results for different viruses), temporal data (epidemiological weeks), and geographic data (reference regions). Classification allowed the database to be structured so that it was easily searchable and ready for subsequent analysis, as summarized in Section 3. Next, the classified data underwent a rigorous pre-processing process. This stage included error cleaning, duplicate removal, format standardization and final validation. For example, dates were standardized, geographic codes were made consistent with official references, and all inconsistencies in the data were resolved to ensure that the final dataset was error-free. Finally, the dataset was published on an open-source platform such as GitHub, available at the link: https://github.com/fbranda/influnet (accessed on 2 February 2025), providing free and transparent access to researchers and public health professionals. Publication on GitHub offered numerous benefits, such as the ability to edit the dataset, tracking changes and making future updates available, and the promotion of collaboration with the scientific community, which can report errors or suggest improvements. In addition, collaboration with Il Sole24Ore, one of Italy’s leading newspapers, has played a key role in making the data easily understandable to citizens, as shown in Figure 2a. Leveraging its expertise in data visualization and communication, Il Sole24Ore has helped present information in a user-friendly format, enabling a wider audience beyond researchers and practitioners to consult and interpret the data effectively [17]. This process has transformed raw, unstructured data into a high-quality resource that is accessible and ready for use in advanced epidemiological analyses, predictive models, and strategic decisions about respiratory disease management in Italy.

The dataset is organized into two main folders: (i) a subfolder called “data-aggregated”, which includes aggregated data from the first report published by ISS (2003–2004 season), and (ii) a subfolder called “flu-season”, which contains data collected for each individual year. Within the “flu-season” folder there is also a subfolder called “bulletins”, where epidemiological and virological reports are stored in PDF format. The preservation of these bulletins is critical, as they contain the original, unaltered reports issued by health authorities, providing an essential reference for understanding the historical context of the data. Preserving these documents also provides a reliable source of information for retrospective analyses.

To facilitate the storage and analysis of the collected data, three primary files have been created, each designed to organize different aspects of the influenza surveillance:

- national_cases.csv and region_cases.csv (Table 1): these files contain data on the total number of reported cases and influenza incidence rates at the national and regional levels. The structure of these files allows easy comparison of trends by geographic area and age group. Key variables included are influenza season, year-week of data collection, region, population statistics, and case incidence rate by age group. The creation of these structured datasets has been critical in tracking seasonal variations and geographic differences in influenza incidence, enabling more informed public health decisions.
- national_typing_subtyping_influenza_viruses.csv (Table 2): this file contains data on the types and subtypes of influenza viruses detected during each influenza season. It includes detailed records of the number of samples collected, sequenced, and the number of viruses detected. This file plays an essential role in identifying circulating strains and understanding the evolution of influenza viruses over time. The development of this data structure ensured that key virological data could be systematically captured and made available for analysis, contributing to viral strain monitoring.
- national_typing_subtyping_influenza_viruses_by_age.csv (Table 3): This file is a more granular version of the previous dataset, which breaks down influenza virus detections by specific age groups (e.g., 0–4, 5–14, 15–64, 65+). Organizing this information into a structured format enables the study of age-specific patterns in influenza virus circulation and helps identify particularly vulnerable populations. The inclusion of age-specific data allows for more targeted public health interventions and a better understanding of the dynamics of virus transmission.

## 3. Results

### 3.1. Epidemiological Profile of Influenza Viruses

Over the past five years, influenza trends in Italy have shown a steady increase. After the COVID-19 pandemic, which was characterized by a more contained viral circulation due to the maintenance of social distancing measures, there has been a gradual upswing in incidence, culminating in particularly pronounced peaks in the most recent seasons. Figure 2 illustrates the weekly trend of influenza-like illness (ILI) incidence at both national and regional levels, emphasizing seasonal fluctuations and variations across age groups. At the national level, Figure 2a shows significant variations across recent seasons. In the 2020–2021 season, the total number of cases was about 60,709, with an average weekly incidence of 1.44%. This value, which is relatively small compared to subsequent years, is probably due to the social distancing measures still in place in the post-pandemic period. In the 2021–2022 season, cases nearly tripled to 174,492, with an average incidence of 3.94%, showing a resurgence of viral activity with the gradual removal of sanitary restrictions. An even more pronounced increase occurred in 2022–2023, with a total of 445,279 cases and a peak incidence exceeding 15.72%. The following season, 2023–2024, recorded 522,209 cases with an average incidence of 8.86%, reaching a high of 18.45%. In the current 2024–2025 season, there were 370,095 total cases with an average incidence of 10.39%, as of 2 February 2025. Looking at the distribution by age group, it is clear that children 0–4 years old were among the most affected, with 45,252 total cases and an average of 2828 cases per week. The 5–14 age group also experienced a significant burden, with 51,827 cases and a higher weekly average of 3239 cases, emphasizing the role of school settings in viral transmission. The 15–64 age group, despite having a lower incidence rate compared to younger children, accounted for the highest absolute number of cases, reaching 219,734 total cases and a weekly average of 13,733 cases. This underscores the substantial impact of influenza on the working population, with potential consequences for absenteeism and workplace productivity. Among the elderly 65+ years old, influenza continues to pose a major health concern, with 53,282 total cases and an average of 3330 cases per week. Although the overall case count in this group is lower than in younger age groups, the heightened risk of severe complications reinforces the need for targeted vaccination strategies and preventive measures.

Regional influenza trends in Italy displayed marked differences in the intensity of the epidemic, with variations shaped by each region’s demographic, geographic, and social characteristics, as illustrated in Figure 2b. Lombardy, the most heavily impacted region, began with 4421 cases in week 42 and saw a sharp rise to 7278 cases by week 5 of 2025. Piedmont followed a similar trajectory, with cases climbing from 1105 in week 42 to 1717 by week 5. The rapid spread in these regions can be attributed to high population density and the significant movement between major cities, which acted as key factors in amplifying the virus transmission. In Central Italy, the increase in cases was more gradual, though still notable. Lazio, for instance, started with 1621 cases in week 42 and reached a peak of 4337 by week 5, showing a steady rise over the period. Emilia-Romagna exhibited a similar pattern, starting with 1171 cases in week 42 and climbing to 2839 by week 5. Although the trends in Central Italy were not as sharp as in the northern regions, they still reflected a clear increase in cases, largely aligning with the return to work and school after the holiday season in early January. In the South, although case numbers remained lower compared to the northern regions, the overall progression of the virus was still notable. Regions like Abruzzo, Apulia, and Sicily experienced marked increases, with Abruzzo seeing cases rise from 792 in week 42 to 2496 by week 5, and Sicily going from 799 to 2124 cases in the same period. Calabria and Basilicata, which did not activate surveillance, saw less visibility in terms of recorded cases but still faced outbreaks as internal mobility increased. The lower population density in the South initially slowed the spread, but the surge in late January and early February, likely driven by increased inter-regional movement, resulted in a significant rise in cases. In general, the influence followed a pattern reflecting the social and economic structure of each region. Areas that were more urbanized and more interconnected were the first to experience significant spikes, while less populated regions saw a smaller increase, although not without impact. Another key factor was the beginning of the year, a time when the reopening of schools and the return to normalcy in work activities contributed to the uptick in cases, encouraging contact between people. Overall, regional flu trends reflect a mix of demographic, social, and structural factors, with a strong correlation between population density, mobility, and the ability of the health care system to respond quickly to the emergency. Differences between the North, Central and South were evident, but all areas experienced some intensification of the spread of the virus, albeit at different times and in different ways.

### 3.2. Virological Profile of Influenza Viruses

**Figure 3:**
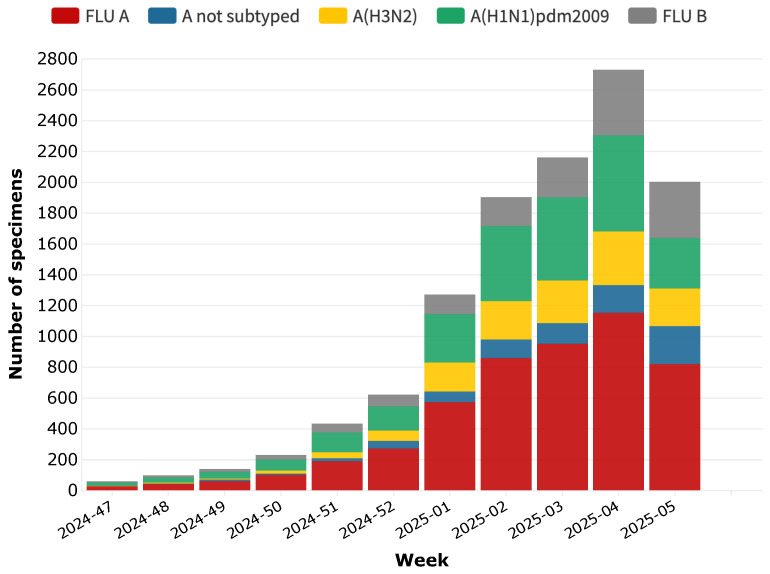
Weekly trend of influenza virus positive samples, by type/subtype, between 18 November 2024 and 2 February 2025.

Figure 3 illustrates the weekly distribution of major respiratory viruses detected during the 2024–2025 influenza season, characterized by the dominance of influenza A, in line with historical trends. Among the detected subtypes, A(H3N2) and A(H1N1)pdm2009 have shown the highest circulation, confirming their persistent role in seasonal influenza outbreaks. Influenza B, although present, has circulated at lower levels than influenza A, showing a delayed increase in cases toward the second half of the flu season.

The detection of influenza viruses showed a steady increase from mid-November 2024, followed by a sharp increase in December, until reaching peak circulation in late December 2024 and early January 2025. This pattern aligns with historical influenza seasonality, where transmission intensifies during the colder months due to increased indoor interactions, school attendance, and weakened immune responses caused by winter conditions. As of early February 2025, a progressive decline in influenza cases has been observed, marking the transition toward the latter phase of the flu season. Notably, the proportion of A(H3N2) cases has been consistently higher than A(H1N1)pdm2009, reinforcing its greater transmissibility and ability to evade pre-existing immunity. This finding is particularly relevant for vaccine effectiveness assessments, as A(H3N2) strains have historically been associated with greater antigenic drift, reducing vaccine-induced protection [18]. The lower circulation of influenza B may be due to cross-immunity effects, lower susceptibility among adults, or differences in population immunity compared to previous seasons [19,20].

**Figure 4:**
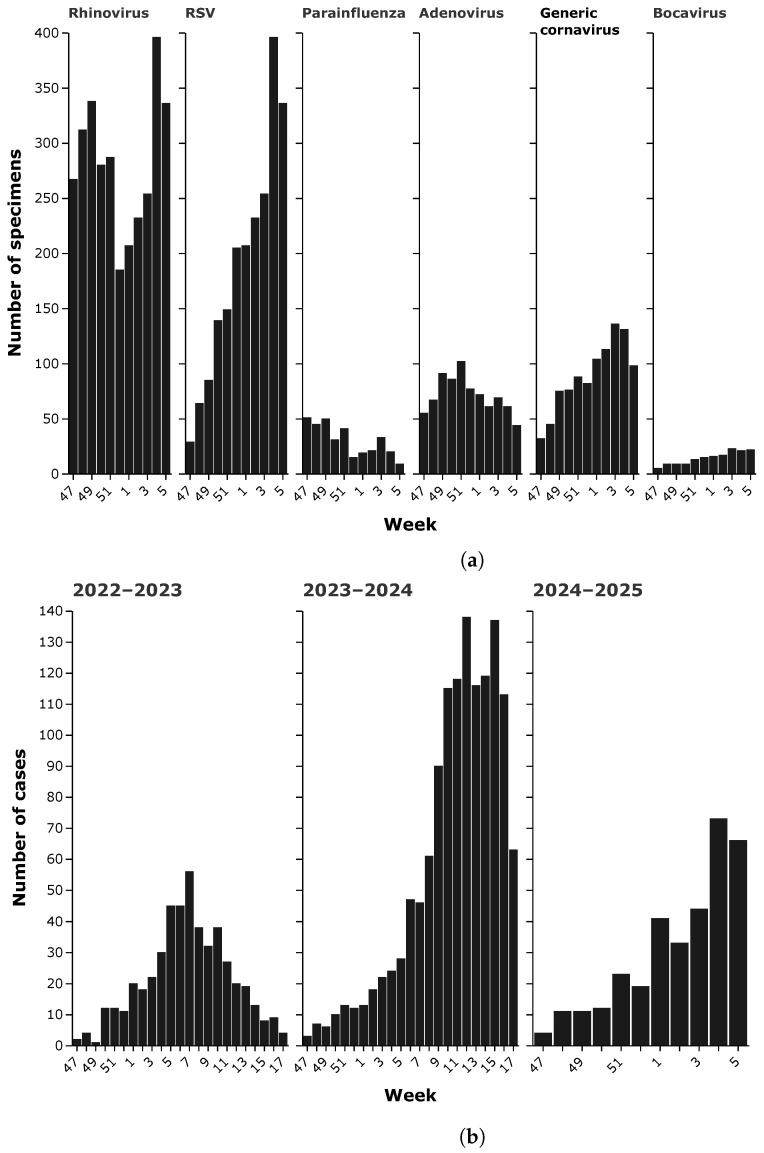
(**a**) Weekly distribution of other respiratory viruses (season 2024–2025). (**b**) Trend of hMPV between November 2022 and 2 February 2025.

In addition to influenza viruses, other respiratory pathogens contribute significantly to the seasonal disease burden, showing distinct but often overlapping temporal patterns (Figure 4a). As of 2 February 2025, Respiratory Syncytial Virus (RSV) showed the highest detection rate among noninfluenza viruses, with a total of 2114 cases reported during the season. Weekly RSV detections increased steadily from mid-November, with 18 cases in week 46, then increased sharply in December and peaked in early January to a high of 396 cases in week 4 of 2025. The observed decline in February suggests a typical seasonality of RSV, with peak circulation slightly later than influenza. Rhinovirus was another major contributor to respiratory infections, with a total of 3341 detections. Unlike RSV, which showed a sharper peak, rhinovirus circulation remained high throughout the season. Weekly detections fluctuated, with an initial peak of 247 cases in week 46, increasing to a peak of 396 cases in week 4, before showing a gradual decrease. The prolonged presence of rhinovirus underscores its role as a year-round pathogen with recurrent peaks, particularly in the colder months when indoor transmission is facilitated. Generic coronaviruses (excluding SARS-CoV-2) accounted for 1003 cases during the season. Weekly detections ranged from 23 to 136 cases, with a gradual increase starting in late November, peaking in weeks 3–4 of 2025 and declining thereafter. The observed trends are consistent with previous reports of seasonal coronaviruses, which tend to circulate at moderate levels along with other respiratory viruses. Adenovirus was detected in 846 cases, with relatively stable weekly numbers throughout the season. Initial detections of 61 cases in week 46 gradually increased to a peak of 102 cases in week 51, followed by minor fluctuations. Unlike influenza or RSV, adenovirus shows a more prolonged and less defined seasonal pattern, probably due to its ability to persist in the population with sporadic outbreaks. Parainfluenza viruses contributed to 374 detections, following a pattern of moderate fluctuations. The highest weekly count was 51 cases in week 47, after which detections remained relatively stable, with occasional increases. The relatively low detection rate compared to RSV or Rhinovirus suggests a more limited role in seasonal respiratory infections, but still contributes to the overall disease burden. Finally, Bocavirus, although less prevalent, was detected in 164 cases. Weekly numbers remained low throughout the season, with detections ranging from 3 to 23 cases per week. Bocavirus is often associated with pediatric respiratory infections, and its relatively low circulation may indicate a lower prevalence in the general population.

Considering hMPV, Figure 4b describes the weekly distribution of hMPV cases in Italy. In the 2022/2023 season, hMPV cases gradually increase, peaking in week 7 of 2023 with 56 cases. Thereafter, a gradual decrease is observed, with a significant drop by week 17 (4 cases). Seasonal trends show a moderate spread, concentrated mainly in the winter months. The 2023/2024 season shows a more rapid and consistent increase than the previous year. Cases reach a very marked peak between weeks 12 and 15 of 2024, with a high of 138 cases in week 12 and 137 cases in week 15. After the peak, cases gradually decrease until week 17 (63 cases). This trend reflects a significant intensification from the previous season, with greater prevalence and a wider and more pronounced curve. In the 2024–2025 season, preliminary data indicate slow growth starting from week 46 (3 cases), with a recorded high of 13 cases in week 51. However, as the season is still in its early stages, it is not possible to outline an overall trend. Comparison of the seasons shows a considerable increase in the spread of the virus in 2023–2024 compared to 2022–2023, with an almost threefold maximum peak (138 vs. 56 cases). Preliminary data for 2024–2025 suggest a similar start to previous seasons, but more data are needed for a complete analysis.

Our analysis emphasize the dynamic nature of seasonal respiratory viruses, with influenza coexisting alongside a wide range of other pathogens. The presence of multiple circulating viruses complicates disease burden assessments, as mixed infections are common and can lead to misdiagnosis or underestimation of disease severity. Continuous genetic and antigenic monitoring of circulating influenza strains remains crucial for vaccine strain selection and public health preparedness. Additionally, the co-detection of RSV, rhinovirus, and hMPV reinforces the need for comprehensive respiratory surveillance programs, particularly in pediatric and elderly populations, where these viruses have the greatest clinical impact.

## 4. Discussion

Recent news about hMPV in China and the DRC in late 2024 highlighted a critical aspect of health crisis management. Regarding the “mystery disease” in the DRC [21], the situation was initially interpreted as a new epidemic, generating much international interest. However, after several weeks of investigation, it was discovered that the symptoms were caused by a combination of already known diseases, such as seasonal viral respiratory infections, falciparum malaria, and acute malnutrition. Although the final diagnosis was correct, the time it took to reach a conclusion drew legitimate criticism. A month to identify the causes of a disease that, for most local doctors, would have been immediately recognizable, highlights the difficulty of responding in a timely manner in settings where resources and technology may be limited. Moreover, this case has raised questions about the speed with which information is shared internationally and the effectiveness of monitoring and response structures in the most vulnerable regions. The lesson that emerges from this episode concerns the importance of not only relying on advanced technologies but also considering the local socio-cultural and epidemiological context.

Another crucial lesson we can learn from these two cases concerns time management and timely response. The speed with which pathogens are identified and how they are treated are critical to avoid the spread of outbreaks. However, haste can also be counterproductive, leading to misdiagnoses and decisions that could prove harmful. In the DRC, data analysis was not fast enough to avoid initial alarmism, but in other contexts, reacting too quickly without adequate verification may have resulted in excessive or inappropriate measures. The key is in the balance: it is necessary to react quickly but in a measured way, supported by solid data and an accurate understanding of the context. Moreover, both cases highlight the importance of clear and evidence-based communication. In an interconnected world, dissemination of information may occur in real time, but this also carries the risk of misinformation and panic. It is essential that health authorities communicate transparently, avoiding both sensationalism and downplaying risks. Communication must be timely, but always supported by a sound scientific basis, so as to reassure the public without concealing any risks. In addition, the role of the media and social platforms is crucial in conveying clear and accurate messages, avoiding the spread of false or alarmist news that could further undermine the health response. This needs to balance alarm and caution calls to mind Aesop’s famous fable of “The Wolf and the Shepherd” [22]. In the fable, the shepherd, in panic, cries “Wolf!” several times to warn the community, but when the wolf comes, no one believes his cry of alarm anymore. Similarly, overly alarmist communication in a health crisis can reduce public confidence and undermine the effectiveness of the response. Excessive early warning without a real threat can be detrimental, as the community may ignore or underestimate the real dangers when they occur.

A crucial aspect of health crisis preparedness is vaccine availability, particularly in low- and middle-income countries (LMICs), where local production capacity is often limited. Vaccine production requires considerable manufacturing capabilities, stringent regulatory oversight, and rigorous quality control processes. For example, as of early 2025, only three human rabies vaccines are prequalified by the WHO [23] for global distribution, reflecting the challenges of ensuring high standards while maintaining adequate supply. Developing local vaccine production remains a complex challenge for many countries, especially in Africa. High costs, lack of infrastructure, and the need for specialized expertise make it difficult to establish sustainable production facilities. Even when local production is initiated, ensuring compliance with international quality standards remains a significant hurdle. The COVID-19 pandemic highlighted these problems, as many LMICs experienced delays in accessing vaccines due to global supply chain constraints and dependence on external manufacturers [24]. To address this disparity, international initiatives such as the GAVI Rabies Vaccine Program, launched in June 2024, play a key role in facilitating access to high-quality vaccines for the least developed countries. Through partnerships with manufacturers, governments, and global health organizations, GAVI provides financial and logistical support to ensure that essential vaccines reach the populations most in need [25]. This program builds on GAVI’s previous efforts in distributing life-saving vaccines, such as those for pneumococcal, rotavirus, and HPV, demonstrating the effectiveness of pooled procurement in reducing costs and increasing supply stability [26]. While GAVI and similar initiatives significantly improve vaccine equity, long-term strategies should focus on strengthening regional manufacturing capacity, improving technology transfer agreements, and promoting public-private partnerships to increase local vaccine manufacturing capacity. The African Union’s Partnership for African Vaccine Manufacturing (PAVM) is an example of an initiative to reduce dependence on external suppliers by developing regional manufacturing hubs [27]. Investing in these approaches will not only improve pandemic preparedness but also strengthen overall health security in vulnerable regions. Additionally, technology transfer and capacity-building efforts [28], are essential for enabling LMICs to produce vaccines locally and sustainably, reducing reliance on global supply chains and ensuring timely access to vaccines during health crises.

Reflecting on the lessons learned from pandemic COVID-19, we need to recognize the vital role of data sharing in mitigating the impact of such crises. The global response to COVID-19 demonstrated both the power and challenges of real-time data exchange. While rapid sharing of epidemiological data [29,30] has helped guide decisions on containment measures, testing, and vaccine development, it has also underscored the need for clear and standardized frameworks for data collection and dissemination.

The Italian surveillance system for respiratory infections represents an established infrastructure that integrates epidemiological and virological data to monitor the spread of influenza and other respiratory viruses. However, access to and interpretation of these data by researchers, health professionals and policy makers have always been a challenge. To address this issue, our research group has developed a visualization and analysis tool based on an open-access database that improves accessibility to surveillance data, enabling exploration of trends, comparison of seasonal patterns, and evaluation of regional variations. This tool is particularly relevant in the Italian context, where the circulation of respiratory viruses is influenced by factors such as regional climatic differences, population density, access to health services, and demographic characteristics. The system enables early identification of anomalies in incidence trends and the assessment of seasonal trends in influenza relative to other respiratory infections, supporting prevention and response strategies. A key advantage of the tool is its ability to provide an intuitive graphical representation of the data, facilitating the interpretation of information not only for the scientific community but also for health authorities and the public. The use of ISS data ensures accurate monitoring, while the use of interactive platforms enables real-time updates and in-depth analysis on specific population groups or geographic areas. In addition, the tool facilitates comparisons between flu seasons, allowing differences in spread patterns and the effectiveness of control strategies adopted to be identified. The application of this system in Italy demonstrates the potential of digital innovation in epidemiological surveillance, contributing to a more effective and targeted response to seasonal epidemics and public health emergencies.

Despite the many advantages of the epidemiological and virological data visualization and analysis tool, there are some limitations that could affect its effectiveness and accuracy in the surveillance of respiratory infections in Italy. One of the main limitations concerns the dependence on the quality and timeliness of the data, as the system relies on information collected by the ISS and other national sources. Any delays in reporting, errors in data collection, or lack of standardization across regions may limit the reliability of analyses and predictions. Another limitation is the geographic coverage and unevenness of surveillance, as some regions may have limited resources to carry out virological testing in a systematic way, resulting in differences in data quality and completeness. Failure to activate surveillance in some areas could affect the national representativeness of the data collected. In addition, the simultaneous circulation of multiple respiratory viruses, such as influenza, RSV, adenovirus, rhinovirus, and seasonal coronavirus, can make it difficult to distinguish individual epidemiological dynamics. The presence of co-infections can complicate analyses, increasing the risk of overestimating or underestimating the impact of a single pathogen.

Several future improvements are planned, including phylogenetic analysis of different influenza strains, which may provide more detailed information on pathogen evolution and transmissibility, improving containment strategies. In addition, enhanced integration with national and regional health information systems will enable more uniform and timely data collection, ensuring greater accuracy in epidemiological analysis. Finally, the optimization of the user interface and the implementation of interactive features for personalized data exploration will be able to make the tool more accessible to a wider audience, facilitating the understanding and use of epidemiological information by researchers, health professionals, and policy makers.

We hope that lessons learned from the recent pandemic experience will guide future efforts to promote international cooperation, improve data transparency, and ensure that the global scientific community is better equipped to respond to future health threats. Only through a shared approach to data management can we build a stronger health system that is ready to respond to new challenges. 

## Figures and Tables

**Table 1:** Data schema for the collection and classification of flu cases, stored in the "national_cases.csv" and "region_cases.csv" files.

Variables	Definition	Format
flu_season	The specific influenza season (e.g., 2022–2023)	String
year_week	Year and week of data collection (e.g., 2022–W01)	String
region	The geographical region for data collection	String
number_healthcare_workers	Number of healthcare workers involved in data collection	String
number_cases	Total number of reported cases	Numeric
population	Total population in the region or area	Numeric
incidence	Incidence rate of influenza cases per population	Numeric
pop_0-4	Population in the 0–4 age group	Numeric
cases_0-4	Number of influenza cases in the 0–4 age group	Numeric
inc_0-4	Incidence rate of influenza cases in the 0–4 age group	Numeric
pop_5-14	Population in the 5–14 age group	Numeric
cases_5-14	Number of influenza cases in the 5–14 age group	Numeric
inc_5-14	Incidence rate of influenza cases in the 5–14 age group	Numeric
pop_15-64	Population in the 15–64 age group	Numeric
cases_15-64	Number of influenza cases in the 15–64 age group	Numeric
inc_15-64	Incidence rate of influenza cases in the 15–64 age group	Numeric
pop_65+	Population in the 65+ age group	Numeric
cases_65+	Number of influenza cases in the 65+ age group	Numeric
inc_65+	Incidence rate of influenza cases in the 65+ age group	Numeric

**Table 2:** Data schema for the collection and classification of respiratory viruses, stored in the file "national_typing_subtyping_influenza_viruses.csv".

Variables	Definition	Format
flu_season	The specific influenza season (e.g., 2022–2023)	String
year_week	Year and week of data collection (e.g., 2022-W01)	String
influenza_viruses	Type of influenza viruses detected (e.g., FLUB, A(H3N2), etc.)	String
number_samples	Total number of samples collected for testing	Numeric
number_sequenced	Number of samples that were sequenced for virus identification	Numeric
number_detections_influenza_viruses	Number of detections of influenza viruses in the samples	Numeric

**Table 3:** Data schema for the collection and classification of respiratory viruses by age, stored in the file "national_typing_subtyping_influenza_viruses_by_age.csv".

Variables	Definition	Format
url_report	URL of the report containing influenza virus data	String
flu_season	The specific influenza season (e.g., 2022–2023)	String
influenza_viruses	Type of influenza viruses detected (e.g., FLUB, A(H3N2), etc.)	String
age_group	Age group of the population (e.g., 0–4, 5–14, 15–64, 65+)	String
number_detections_influenza_viruses	Number of detections of influenza viruses in the samples	Numeric

## Data Availability

The data presented in this study are available at https://github.com/fbranda/influnet, accessed on 2 February 2025. These data were derived from the weekly bulletins published by ISS, which are publicly available at https://respivirnet.iss.it/pagine/rapportoInflunet.aspx, accessed on 2 February 2025.
